# Opa1 Prevents Apoptosis and Cisplatin-Induced Ototoxicity in Murine Cochleae

**DOI:** 10.3389/fcell.2021.744838

**Published:** 2021-09-21

**Authors:** Tingting Dong, Xuejie Zhang, Yiqing Liu, Shan Xu, Haishuang Chang, Fengqiu Chen, Lulu Pan, Shaoru Hu, Min Wang, Min Lu

**Affiliations:** ^1^Biobank of Ninth People’s Hospital, Shanghai Jiao Tong University School of Medicine, Shanghai, China; ^2^Shanghai Ninth People’s Hospital, Shanghai Jiao Tong University School of Medicine, Shanghai, China; ^3^Shanghai Ninth People’s Hospital, Shanghai Institute of Precision Medicine, Shanghai Jiao Tong University School of Medicine, Shanghai, China; ^4^Shanghai Key Laboratory for Prevention and Treatment of Bone and Joint Diseases, Department of Orthopaedics, Ruijin Hospital, Shanghai Institute of Traumatology and Orthopaedics, Shanghai Jiao Tong University School of Medicine, Shanghai, China

**Keywords:** OPA1, mitochondria, cisplatin, apoptosis, ototoxicity

## Abstract

Optic atrophy1 (OPA1) is crucial for inner mitochondrial membrane (IMM) fusion and essential for maintaining crista structure and mitochondrial morphology. Optic atrophy and hearing impairment are the most prevalent clinical features associated with mutations in the *OPA1* gene, but the function of OPA1 in hearing is still unknown. In this study, we examined the ability of Opa1 to protect against cisplatin-induced cochlear cell death *in vitro* and *in vivo*. Our results revealed that knockdown of *Opa1* affects mitochondrial function in HEI-OC1 and Neuro 2a cells, as evidenced by an elevated reactive oxygen species (ROS) level and reduced mitochondrial membrane potential. The dysfunctional mitochondria release cytochrome c, which triggers apoptosis. *Opa1* expression was found to be significantly reduced after cell exposed to cisplatin in HEI-OC1 and Neuro 2a cells. Loss of Opa1 aggravated the apoptosis and mitochondrial dysfunction induced by cisplatin treatment, whereas overexpression of *Opa1* alleviated cisplatin-induced cochlear cell death *in vitro* and *in explant*. Our results demonstrate that overexpression of *Opa1* prevented cisplatin-induced ototoxicity, suggesting that Opa1 may play a vital role in ototoxicity and/or mitochondria-associated cochlear damage.

## Introduction

Mitochondria are responsible for numerous vital cell functions, such as respiration, oxidative phosphorylation (OXPHOS), calcium homeostasis, and apoptotic signaling. These highly dynamic organelles continually modify their shape and undergo fusion and fission in order to maintain their morphology and activity (Hoppins et al., 2007; Rogers et al., 2017). Mitofusin 1 and 2 (Mfn1 and Mfn2) are crucial for outer mitochondrial membrane (OMM) fusion (Santel and Fuller, 2001; Chen et al., 2003), and the dynamin-related GTPase, OPA1, is essential for IMM fusion (Olichon et al., 2002). Dynamin-related protein 1 (Drp1) is a cytosolic protein that is required for mitochondrial fission. The recruitment of Drp1 is regulated by the OMM proteins, mitochondrial fission factor (Mff), mitochondrial division (Mid) 49, Mid51, and mitochondrial fission protein (Fis1) (Loson et al., 2013; Palmer et al., 2013). Mitochondrial fusion and fission are vital processes for cellular functions. Numerous studies have drawn connections between abnormal mitochondrial morphology and various diseases, including neurodegenerative diseases, cardiovascular diseases, and cancers (Mishra and Chan, 2014; Liu L. et al., 2016; He et al., 2017; Li A. et al., 2018; Gao et al., 2019; Zhang et al., 2019; Zhong et al., 2020; Fu et al., 2021b).

*OPA1*, which is localized at chromosome 3q28, has been identified as the causative gene of Dominant Optic Atrophy (DOA) (Alexander et al., 2000; Delettre et al., 2000). The *OPA1* mRNA has eight alternatively spliced isoforms (Delettre et al., 2001), while the OPA1 protein exists in two forms, the membrane-bound long-OPA1 (L-OPA1) and the soluble short-OPA1 (S-OPA1). Both are required for mitochondrial fusion (Olichon et al., 2002; Ishihara et al., 2006). In addition to its function in IMM fusion, OPA1 is crucial for the maintenance of crista structure and mitochondrial morphology (Frezza et al., 2006; Patten et al., 2014). Opa1 depletion was associated with mitochondrial fragmentation, crista disorganization, and cytochrome c redistribution (Frezza et al., 2006). In contrast, *Opa1* overexpression was associated with: normally shaped cristae; alterations in the responses of multiple tissues to apoptotic, necrotic, and atrophic stimuli; and phenotypic rescue of mitochondrial diseases in mice (Civiletto et al., 2015; Varanita et al., 2015). Opa1 also contributes to safeguarding mtDNA integrity, preserving mtDNA function in the face of mutations (Chen et al., 2010), and supporting cellular adaptation to metabolic demand (Patten et al., 2014). Opa1 dysfunction has been linked to ROS overproduction and unbalanced redox homeostasis (Yarosh et al., 2008; Chen et al., 2012).

In the clinic, about 60–70% of DOA cases are associated with pathogenic mutations of *OPA1* (Alexander et al., 2000; Delettre et al., 2000). DOA is characterized by destruction of retinal ganglion cells (RGCs) and the optic nerve (Kjer, 1959), deafness, and chronic progressive external ophthalmoplegia (Treft et al., 1984; Meire et al., 1985). Hearing loss usually follows the onset of visual symptoms (Leruez et al., 2013). Although OPA1 is highly expressed in the retina, it is broadly expressed in multiple tissues. In cochlear tissue, OPA1 is expressed in both hair cells and ganglion cells. Unlike ganglion cells, which express OPA1 at birth, OPA1 expression in the organ of Corti increases after birth and approaches the mature expression level during the onset of hearing (Bette et al., 2007). This suggests that OPA1 is crucial for the function of hair cells and ganglion cells in the inner ear.

In this study, we investigated the potential effect of Opa1 to protect against cisplatin-induced apoptosis *in vitro* and *in vivo*. Our results indicated that loss of Opa1 caused mitochondrial dysfunction and thereby triggered apoptosis in HEI-OC1 and Neuro 2a cells, an auditory hair cell line and a neural cell line. Cisplatin treatment markedly reduced *Opa1* expression in HEI-OC1 and Neuro 2a cells. In the two tested cell lines, Opa1 depletion aggravated the cisplatin-induced apoptosis and mitochondrial dysfunction, whereas *Opa1* overexpression partially prevented cisplatin-induced apoptosis. Histological analyses were performed using cisplatin-treated organotypic cochlear cultures, and our results showed that Opa1 overexpression decreased cisplatin-induced cell death, indicating Opa1 is a potential therapeutics gene in cisplatin induced auditory impairment.

## Materials and Methods

### Cell Culture

Murine HEI-OC1 cells were grown in DMEM supplemented with 10% heat-inactivated FBS and maintained at 33°C with 10% CO2. Murine Neuro 2a cells were grown in the same medium at 37°C with 5% CO2. HEI-OC1 and Neuro 2a cells were incubated in 96-well plates for cell viability assessment or 6-well plates for flow cytometry, transmission electron microscopy, and immunostaining. siRNA or plasmids were transfected after cells were grown to 60% confluence. siRNA was transfected using LipoRNAiMax (Invitrogen). Plasmids were transfected into Neuro 2a cells using Lipo2000 (Invitrogen, 11668-019) and into HEI-OC1 cells using Lipo3000 (Invitrogen, L30000015), with the relevant reagents diluted in Opti-MEM (Gibco). The cells were then treated with or without cisplatin for 24 h. For cell viability detection, HEI-OC1 or Neuro 2a cells were transfected with siRNA or plasmids, and then treated with or without cisplatin for 24 h, unless indicated the timepoint in the picture. For qRT-PCR analyses, HEI-OC1 or Neuro 2a cells were grown in a 6-well plate to 70–80% confluence, treated with siRNA, plasmids or cisplatin for 24 h. For Western blot, HEI-OC1 or Neuro 2a cells were grown in a 6-well plate to 70–80% confluence, treated with siRNA, plasmids or cisplatin for 48 h. For flow cytometry, transmission electron microscopy, and immunostaining, cells were transfected with siRNA or treated with or without cisplatin for 24 h. For almost all the experiments both HEI-OC1 and Neuro 2a cells were used. Several experiments (Mitochondria morphology detection after Opa1 knockdown and cisplatin treatment; Cytochrome C release analysis; TEM; Bcl-2 protein expression; and DCF level after cisplatin treatment) were only used HEI-OC1 cells.

### Cell Viability Assessment

HEI-OC1 and Neuro 2a cells were plated in 96-well plates (1 × 10^5^ cells/well) and incubated with 100 μL drug-supplemented DMEM. After 24, 48, or 72 h, cell viability was measured using a Cell Counting Kit-8 (CCK-8; Dojindo) according to the manufacturer’s instructions. The optical density at 450 nm (OD450 nm) was measured using a Tecan Spark multimode microplate reader.

### Flow Cytometry

To test the ROS level and mitochondrial membrane potential (MMP), cells were collected, washed thrice with PBS, and stained using DCFH-A (Sigma, D6883; 10 mM in DMSO, 1:1,000) or TMRM (Invitrogen, 134361; 1:1,000) at 37°C for 30 min, and then counterstained with DAPI (Invitrogen,D357; 1:1,000). Apoptosis was assessed with apoptosis detection kit (BioLegend, San Diego, CA, United States). Briefly, cells were collected, washed thrice with PBS, suspended with 500 μL binding buffer, and stained with Annexin V-FITC and propidium iodide following the manufacturer’s instructions. Stained cells were analyzed using a flow cytometer and data were processed with the FlowJo software (FlowJo, LLC, Ashland, OR, United States).

### Western Blot Analysis

Collected cells were lysed with RIPA buffer (Thermo Fisher Scientific, 89900) containing a complete protease inhibitor cocktail (Roche) at 4°C for 20 min with shaking, and then centrifuged at 4°C for 20 min at 13,000 rpm. The supernatant was collected, the total proteins were quantified, and 10 μg of total protein from each sample was boiled for 10 min in sample buffer (BioRad, #161-0737), resolved by SDS-PAGE, and transferred to a PVDF membrane. The membrane was blocked with TBST containing 5% skim milk, incubated with primary antibodies in TBST containing 5% skim milk, and incubated with HRP-conjugated secondary antibodies. The results were visualized with ECL reagents. The following primary antibodies were used: mouse anti-Opa1 (BD, 612606), rabbit anti-Hsp60 (CST, 12165), rabbit anti-Bcl2 (CST,3498), and rabbit anti-β-actin (CST, 4967S).

### Quantitative Real-Time Reverse Transcription Polymerase Chain Reaction

Total RNA was extracted from cultured cells using a commercially available kit (Qiagen, #74136) with the optional DNase digestion step. The RNA was reverse transcribed with an Evo M-MLV RT kit with gDNA Clean for qPCR (Accurate Biotechnology, China) and qRT-PCR was performed using a Roche LightCycler 480 with a SYBR Green Premix Pro Taq HS qPCR kit (Accurate Biotechnology) and the following cycling conditions: 95°C for 5 min followed by 45 cycles of 95°C for 10 s, 60°C for 10 s, and 72°C for 10 s. The relative level of each target gene was normalized to that of endogenous *Rpl19* and calculated using the comparative Ct (ΔΔCt) method. The sequences of the utilized primers were as follows: 5′- TGGAAAATGGTTCGAGAGTCAG-3′ (forward) and 5′- CATTCCGTCTCTAGGTTAAAGCG-3′ (reverse) for *Opa1*; 5′-ACGGAGGCTGGGATGCCTTTG-3′ (forward) and 5′-AGTGATGCAGGCCCCGACCA-3′ (reverse) for *Bcl2*; and 5′-ACCTGGATGAGAAGGATGAG-3′ (forward) and 5′-ACCTTCAGGTACAGGCTGTG-3′ (reverse) for *Rpl19*.

### Transmission Electron Microscopy

HEI-OC1 cells were collected, placed in 2.5% glutaraldehyde, and incubated at room temperature for 20 min and then at 4°C for 1 h. The cells were post-fixed with 1% osmium tetroxide for 2 h at room temperature, dehydrated in a graded alcohol series, and infiltrated in a graded alcohol series containing eopn812. The samples were double-stained with uranyl acetate followed by lead citrate and examined with a 120 kV transmission electron microscope (FEI Talos L120C).

### Organotypic Cochlear Cultures

Organotypic cochlear cultures were generated from the cochlea of postnatal day 1–2 (P1–P2) mouse pups of both sexes. Briefly, mouse pups were sacrificed by decapitation and dissected in HBSS supplemented with HEPES. The basal membrane was isolated, plated in a glass-bottom dish with DMEM/F12 medium containing 1 × N2 (Sigma, A1370701) and 1 × B27 (Sigma, 17504044), and incubated overnight at 37°C with 5% CO2. For immunostaining, the basal membrane cells were simultaneously transfected with *Opa1* plasmid (p*Opa1*) or siRNA and treated with 60 μM cisplatin for 24 h. Control cultures were treated with cisplatin alone for 24 h or left untreated.

### Immunofluorescence Assay

HEI-OC1 and Neuro 2a cells were collected and rinsed thrice in phosphate-buffered saline (PBS) for 10 min per wash. The cells were fixed in 4% paraformaldehyde (PFA) for 30 min and incubated overnight at 4°C with anti-cleaved caspase-3 (CST, 9661; rabbit, 1:400). The cells were rinsed thrice with PBS, incubated with Alexa Fluor 488 donkey anti-rabbit IgG antibody (Invitrogen, 1:400) for 60 min at room temperature, rinsed thrice in PBS, and stained with DAPI (YEASEN, 36308ES20). Alternatively, cells were incubated with MitoTracker (Invitrogen; M7510, 1:10,000) at 37°C for 30 min, rinsed thrice in PBS, fixed in 4% PFA for 30 min, and then either directly sealed with DAPI or incubated overnight at 4°C with anti-cytochrome c (CST, 11940; rabbit, 1:100), rinsed thrice in PBS, incubated with Alexa Fluor 488 donkey anti-rabbit IgG antibody (Invitrogen; 1:400) for 60 min at room temperature, rinsed thrice in PBS, and then sealed with DAPI.

The basal membrane cells were collected, rinsed thrice in PBS for 10 min per rinse, fixed in 4% PFA for 30 min, incubated overnight at 4°C with MYO 7A (proteus biosciences; 1:500), rinsed thrice in PBS, incubated with Alexa Fluor 488 donkey anti-rabbit IgG antibody (Invitrogen; 1:400) for 60 min at room temperature, rinsed thrice in PBS, and sealed with DAPI.

### Statistical Analysis

Data are presented as mean ± standard errors of the mean (SEM). Experiments were performed in triplicate and repeated three times. Statistical significance was assessed with *t*-test, one-way ANOVA, or two-way ANOVA, as applied using the GraphPad Prism 9.0 software (GraphPad Software). A value of *p* < 0.05 was considered statistically significant.

## Results

### Knockdown of *Opa1* in HEI-OC1 and Neuro 2a Cells by siRNA

**FIGURE 1:**
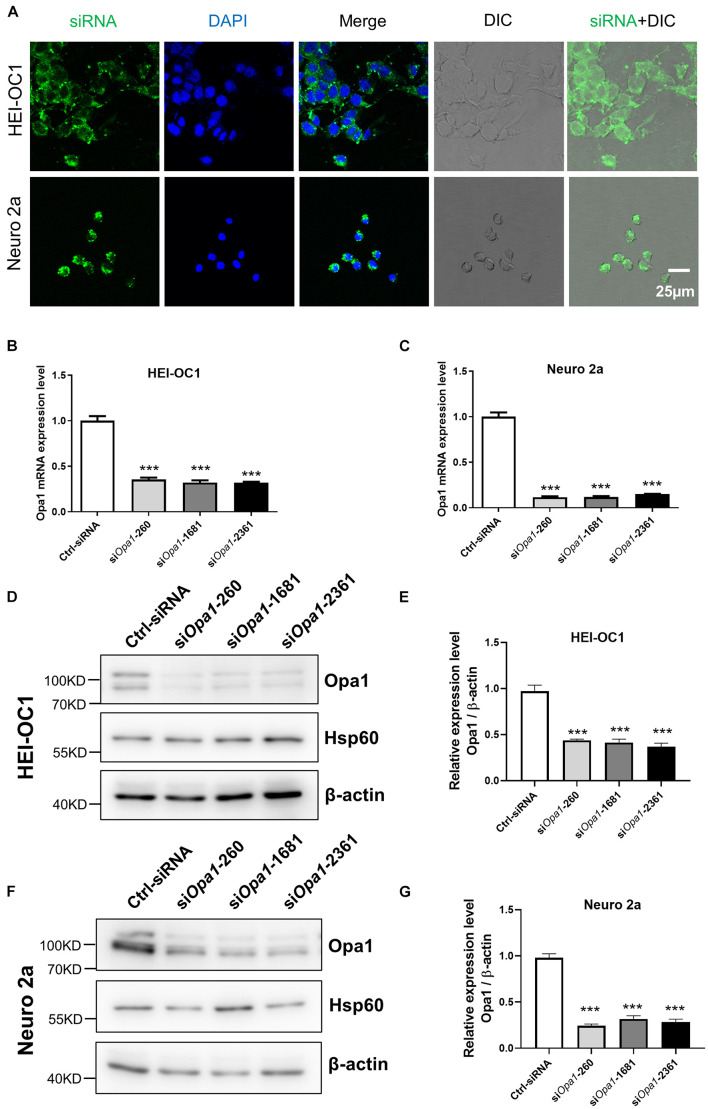
Depletion of Opa1 in HEI-OC1 and Neuro 2a cells. **(A)** Representative confocal images of HEI-OC1 and Neuro 2a cells after siRNA transfection for 24 h, showing transfection efficiency. DIC: Differential interference contrast. Quantification of *Opa1* mRNA expression levels in HEI-OC1 **(B)** and Neuro 2a **(C)** cells transfected with si*Opa1* for 24 h. In this and all following graphs: the levels of *Opa1* mRNA were normalized to that of mouse rpl19; error bars represent standard error of the mean (SEM); and experiments were performed in triplicate and repeated three times. ***p < 0.001 (one-way ANOVA with Dunnett’s multiple comparisons test). Representative Western blot images **(D)** and quantification **(E)** of *Opa1* expression in HEI-OC1 cells transfected with si*Opa1* for 48 h. In this and all following graphs, the protein levels of Opa1 are normalized to that of β-actin. ***p < 0.001 (one-way ANOVA with Dunnett’s multiple comparisons test). Representative Western blot images **(F)** and quantification **(G)** of *Opa1* expression in Neuro 2a cells transfected with si*Opa1* for 48 h. ***p < 0.001 (one-way ANOVA with Dunnett’s multiple comparisons test).

To study the role of Opa1 in the inner ear, we used siRNA transfection to assess the effect of *Opa1* knockdown in HEI-OC1 and Neuro 2a cells, which were chosen to represent two important cell types of the inner ear: hair cells and spiral ganglion neurons. In the latter case, we selected the most common used neuronal cell line because there was no specific cell line available for spiral ganglion neurons. The transfection efficiency was monitored by transfecting cells with fluorescence-tagged sham siRNA. Almost all HEI-OC1 and Neuro 2a cells were fluorescence-positive at 24 h post-transfection (Figure 1A). We designed three different mouse *Opa1* siRNA (si*Opa1*-260, si*Opa1*-1681, si*Opa1*-2361) and analyzed their effects in HEI-OC1 and Neuro 2a cells compared to those of a control scramble siRNA (Ctrl-siRNA). At 24 h after si*Opa1* transfection, *Opa1* mRNA expression was reduced by 70% in HEI-OC1 cells (Figure 1B) and by 90% in Neuro 2a cells (Figure 1C). To test whether there was a corresponding reduction in the protein expression of Opa1, we performed Western blot analysis at 48 h after si*Opa1* transfection. All three of the *Opa1* siRNA were found to significantly decrease the Opa1 protein levels in the two cell lines (Figures 1D,F). Quantification revealed that Neuro 2a cells displayed less Opa1 protein expression than HEI-OC1 cells (Figures 1E,G), which was consistent with the trends in their mRNA expression levels (Figures 1B,C). These results indicate that Opa1 could be successfully suppressed by the designed siRNA in HEI-OC1 and Neuro 2a cells. For the following experiments, si*Opa1*-260 was used for *Opa1* knockdown subsequently.

### Knockdown of *Opa1* Impairs Mitochondrial Function

**FIGURE 2:**
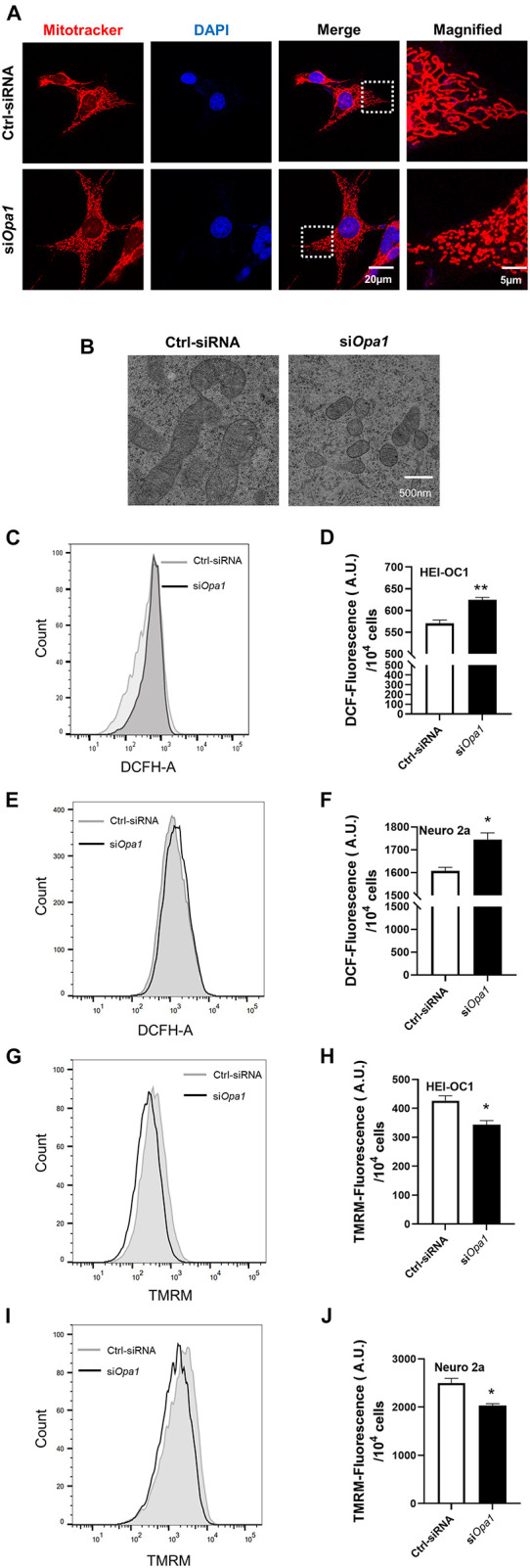
Loss of Opa1 leads to mitochondrial dysfunction in HEI-OC1 and Neuro 2a cells. **(A)** Representative confocal microscopic images of MitoTracker staining at 24 h after siRNA transfection of HEI-OC1 cells. Right panels show magnified views of the areas marked in the left panels. **(B)** HEI-OC1 cells were transfected with scramble (Ctrl) siRNA or si*Opa1* for 24 h and fixed, and thin sections of cells were visualized by transmission electron microscopy. **(C)** Intracellular ROS level was measured by flow cytometric analysis using DCFH-DA in HEI-OC1 cells transfected with siRNA for 24 h. **(D)** Quantitative histograms of the mean fluorescence intensity of DCFH-DA in HEI-OC1 cells. **p < 0.01 (non-parametric Mann-Whitney *t*-test). **(E,F)** Representative flow cytometric plot **(E)** and quantitative histograms **(F)** of the mean fluorescence intensity of DCFH-DA in Neuro 2a cells transfected with siRNA for 24 h. *p < 0.05 (non-parametric Mann-Whitney *t*-test). **(G)** The mitochondrial membrane potential of HEI-OC1 cells was measured by flow cytometric analysis using TMRM fluorescence at 24 h after siRNA transfection. **(H)** Bar graph shows the relative TMRM mean intensity in HEI-OC1 cells. *p < 0.05 (non-parametric Mann-Whitney *t*-test). Representative flow cytometric plot **(I)** and quantitative histograms **(J)** of the relative TMRM mean intensity in Neuro 2a cells. *p < 0.05 (non-parametric Mann-Whitney *t*-test).

To study the cellular function of Opa1 in HEI-OC1 and Neuro 2a cells, we first monitored mitochondrial morphology by electron microscope and MitoTracker staining. MitoTracker staining and Electron microscopic analysis of HEI-OC1 cells transfected with Ctrl-siRNA revealed mainly elongated mitochondria (Figure 2A), with some tubular mitochondria (Figure 2B). HEI-OC1 cells transfected with *Opa1* siRNA for 24 h exhibited highly fragmented, dot-like mitochondria, as assessed by MitoTracker staining (Figure 2A) and electron microscopy (Figure 2B). In contrast, mitochondria of Neuro 2a cells displayed fragmented shapes prior to siRNA transfection (data not shown), and there was no further difference of mitochondria shape between Ctrl-siRNA- and si*Opa1*-transfected Neuro 2a cells under MitoTracker staining (data not shown). To assess whether Opa1 depletion affected mitochondrial function, we transfected the cells with siRNA for 24 h and measured the ROS level and mitochondrial membrane potential (MMP, ΔΨ_m_) using DCFH-DA and TMRM, respectively. HEI-OC1 cells lacking Opa1 exhibited an increased ROS level (Figures 2C,D) and reduced MMP (Figures 2G,H) compared with control cells. Despite displaying no alteration of mitochondrial shape, *Opa1*-silenced Neuro 2a cells displayed a higher ROS level (Figures 2E,F) and lower MMP (Figures 2I,J) compared with control cells. These findings indicate that Opa1 depletion affects mitochondrial function in HEI-OC1 and Neuro 2a cells, independent of its contribution to mitochondrial formation.

### Silencing of *Opa1* Increases Vulnerable to Apoptosis

**FIGURE 3:**
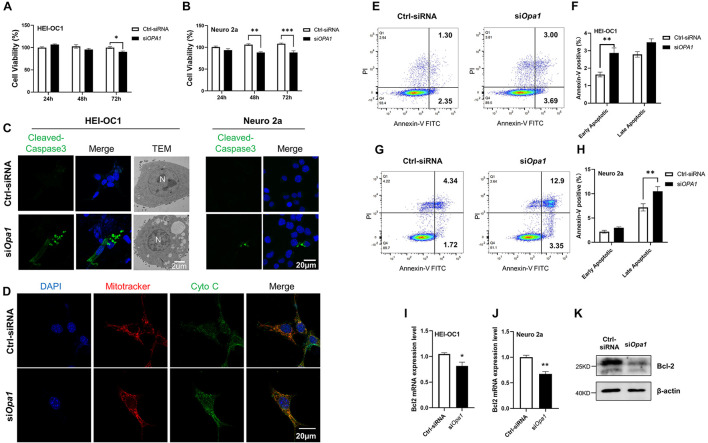
Lacking Opa1 triggers apoptosis in HEI-OC1 and Neuro 2a cells. **(A,B)** CCK-8 was used to assess the cell viability of HEI-OC1 **(A)** and Neuro 2a **(B)** cells cultured for 24, 48, and 72 h after transfection with scramble siRNA (Ctrl) or si*Opa1*. *p < 0.05, **p < 0.01, ***p < 0.001 (two-way ANOVA). **(C)** Representative confocal microscopic images of cleaved caspase-3 staining at 24 h after siRNA transfection of HEI-OC1 and Neuro 2a cells. Representative transmission electron microscopic images of HEI-OC1 cells at 24 h after siRNA transfection. **(D)** Representative confocal images of HEI-OC1 cells co-stained for mitochondria (MitoTracker) and cytochrome c at 24 h after siRNA transfection. **(E)** Representative flow cytometric plots generated from HEI-OC1 cells stained with Annexin V-FITC/PI to detect apoptosis at 24 h after transfection with scramble siRNA (Ctrl) or si*Opa1*. **(F)** The percentages of early and late apoptotic cells were compared between Ctrl-siRNA- and si*OPA1*-transfected HEI-OC1 cells. **p < 0.01 (two-way ANOVA). FITC, fluorescein isothiocyanate; PI, propidium iodide. **(G)** Representative flow cytometric plots generated from Neuro 2a cells stained with Annexin V-FITC/PI to detect apoptosis at 24 h after transfection with scramble siRNA (Ctrl) or si*Opa1*. **(H)** The percentages of early and late apoptotic cells were compared between Ctrl-siRNA- and si*OPA1*-transfected Neuro 2a cells. **p < 0.01 (two-way ANOVA). **(I,J)** Quantification of Bcl2 mRNA expression levels in HEI-OC1 (I) and Neuro 2a **(J)** cells transfected with siRNA for 24 h. *p < 0.05, **p < 0.01 (non-parametric Mann-Whitney *t*-test). **(K)** Representative Western blot images showing Bcl2 protein levels in Neuro 2a cells transfected with siRNA for 48 h.

Next we sought to identify the cellular changes that arise from *Opa1* silencing. At 24 h post-transfection, the cell numbers were comparable between si*Opa1*- and Ctrl-siRNA-transfected HEI-OC1 and Neuro 2a cells (Figures 3A,B, left). At 48 h post-transfection, Opa1-depleted Neuro 2a cells displayed a significant increase in cell death, whereas Opa1-depleted HEI-OC1 cells did not (Figures 3A,B, middle). At 72 h post-transfection, Opa1-depleted Neuro 2a and HEI-OC1 cells both exhibited significant increases in cell death (Figures 3A,B, right). To assess whether cells underwent apoptosis upon *Opa1* knockdown, we stained siRNA-transfected cells for cleaved caspase-3. Cleaved caspase-3 staining was evident in HEI-OC1 and Neuro 2a cells transfected with si*Opa1* (Figure 3C), indicating that apoptosis was activated upon Opa1 depletion. In agreement with this increased caspase-3 activation, transmission electron microscopy revealed the presence of apoptotic cells in cultures of Opa1-depleted HEI-OC1 cells (Figure 3C). We postulated that loss of Opa1 decreased the MMP (Figures 2E,I) and led to cytochrome c leakage, thereby increasing caspase-3 activation. In support of this proposal, staining experiments revealed that cytochrome c and mitochondria were colocalized in HEI-OC1 cells transfected with Ctrl-siRNA, as reflected by an orange color representing the merging of green (cytochrome c) and red (MitoTracker) fluorescence signals (Figure 3D). In *Opa1*-knockdown cells, in contrast, we observed largely non-overlapping green and red signals that suggested the cytochrome c had been released from the mitochondria (Figure 3D). To further confirm that cells underwent apoptosis following si*Opa1* transfection, we preformed Annexin V/PI staining and flow cytometric analysis. Annexin V staining showed that knockdown of *Opa1* induced more early apoptotic cells in HEI-OC1 cultures (Figures 3E,F) and more late apoptotic cells in Neuro 2a cultures (Figures 3G,H). This finding is consistent with our observation that Opa1 depletion led to earlier cell death in Neuro 2a cells than in HEI-OC1 cells (Figures 3A,B). Bcl family proteins, such as Bcl-2, regulate the release of cytochrome c through the OMM (Adams and Cory, 2007). To assess whether Bcl2 was involved in the apoptosis induced by *Opa1* knockdown, we detected Bcl2 expression in cells transfected with siRNA for 24 h. After Opa1 depletion, the mRNA level of Bcl2 was markedly reduced in both HEI-OC1 and Neuro 2a cells (Figures 3I,J), and the protein level of Bcl2 was reduced in Neuro 2a cells (Figure 3K).

### Knockdown of *Opa1* Aggravates Cisplatin-Induced Mitochondrial Dysfunction and Apoptosis

**FIGURE 4:**
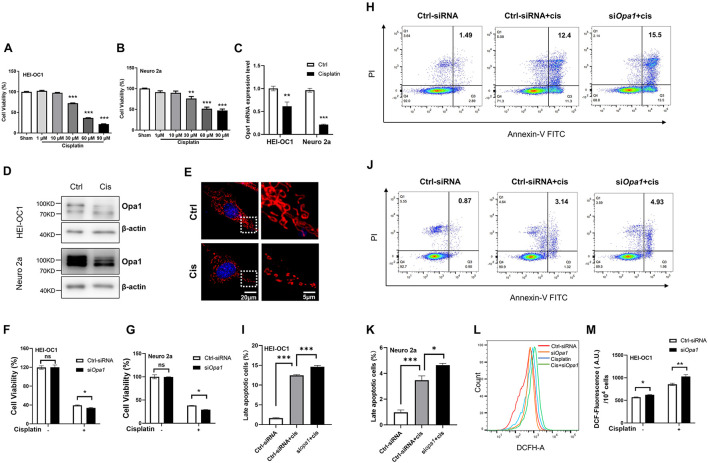
Knockdown of *Opa1* aggravates cisplatin-induced apoptosis and mitochondrial dysfunction in HEI-OC1 and Neuro 2a cells **(A,B)** CCK-8 was used to assess cell viability in HEI-OC1 **(A)** and Neuro 2a **(B)** cells treated with the indicated doses of cisplatin for 24 h. **p < 0.01, ***p < 0.001 (one-way ANOVA with Dunnett’s multiple comparisons test). **(C)** Quantification of Opa1 mRNA expression levels in HEI-OC1 and Neuro 2a cells treated with cisplatin for 24 h. **p < 0.01, ***p < 0.001 (two-way ANOVA). **(D)** Representative Western blot images of *Opa1* expression in HEI-OC1 and Neuro 2a cells treated with cisplatin for 24 h. **(E)** Representative confocal microscopic images of MitoTracker staining at 24 h after cisplatin treatment of HEI-OC1 cells. Right panels show magnified views of the areas marked in the left panels. **(F,G)** CCK-8 was used to assess cell viability in HEI-OC1 **(F)** and Neuro 2a **(G)** cells at 24 h after siRNA transfection with or without cisplatin treatment. N.S., non-significant, *p < 0.05 (two-way ANOVA). **(H)** Representative flow cytometric plots were generated from HEI-OC1 cells stained with Annexin V-FITC/PI to detect apoptosis at 24 h after siRNA transfection with or without cisplatin treatment. **(I)** The percentage of late apoptotic cells in each group. ***p < 0.001 (one-way ANOVA with Dunnett’s multiple comparisons test). **(J)** Representative flow cytometric plots were generated from Neuro 2a cells stained with Annexin V-FITC/PI to detect apoptosis at 24 h after siRNA transfection with or without cisplatin treatment. **(K)** The percentage of late apoptotic cells in each group. *p < 0.05, ***p < 0.001 (one-way ANOVA with Dunnett’s multiple comparisons test). **(L)** Intracellular ROS levels were measured by flow cytometric analysis of HEI-OC1 cells stained with DCFH-DA at 24 h after siRNA transfection with or without cisplatin treatment. **(M)** Quantitative histograms of the mean fluorescence intensity of DCFH-DA in HEI-OC1 cells. *p < 0.05, **p < 0.01 (two-way ANOVA).

Serious side effects, such as ototoxicity, are associated with the use of cisplatin. Several studies demonstrated that cisplatin-induced cytotoxicity is closely related to mitochondrial dysfunction such as that signaled by ROS generation (van Gisbergen et al., 2015; de Sa Junior et al., 2017). Increased ROS alters the MMP and induces damage in the respiratory chain, thereby triggering apoptosis. To identify a suitable concentration of cisplatin that could induce cellular damage in our system, we exposed HEI-OC1 and Neuro 2a cells to various concentrations of cisplatin. After incubation with 60 μM cisplatin for 24 h, cell death was observed in 60% of HEI-OC1 cells and 50% of Neuro 2a cells (Figures 4A,B). Interestingly, *Opa1* mRNA expression was reduced in HEI-OC1 and Neuro 2a cells exposed to cisplatin for 24 h (Figure 4C). Consistent with mRNA level, Opa1 protein level also decreased after cisplatin treatment in the two cell lines (Figure 4D). According to MitoTracker staining, HEI-OC1 cells treated with cisplatin for 24 h exhibited highly fragmented, dot-like mitochondria (Figure 4E), which is similar to the morphology of mitochondrion in HEI-OC1 cells with *Opa1* knockdown (Figure 2A). Treatment with si*Opa1* for 24 h did not cause cell death in HEI-OC1 or Neuro 2a cells, but loss of Opa1 significantly worsened cell survival upon cisplatin treatment (Figures 4F,G). To confirm these cell viability results, we performed Annexin V/PI staining followed by flow cytometric analysis. Annexin V staining showed that cisplatin treatment for 24 h induced ∼12% late apoptotic cells in HEI-OC1 cells and ∼3.5% late apoptotic cells in Neuro 2a cells (Figures 4H–K). Silencing of *Opa1* additively enhanced the proportions of late apoptotic cells in cisplatin-treated HEI-OC1 and Neuro 2a cell cultures (Figures 4I,K). Moreover, the mitochondrial dysfunction caused by cisplatin treatment was worsened by Opa1 depletion, as evidenced by increased ROS generation in HEI-OC1 cells (Figures 4L,M). These results indicate that Opa1 depletion exacerbates the mitochondrial dysfunction and apoptosis induced by cisplatin in both HEI-OC1 and Neuro 2a cells.

### Opa1 Upregulation Suppresses Cisplatin-Induced Apoptosis

**FIGURE 5:**
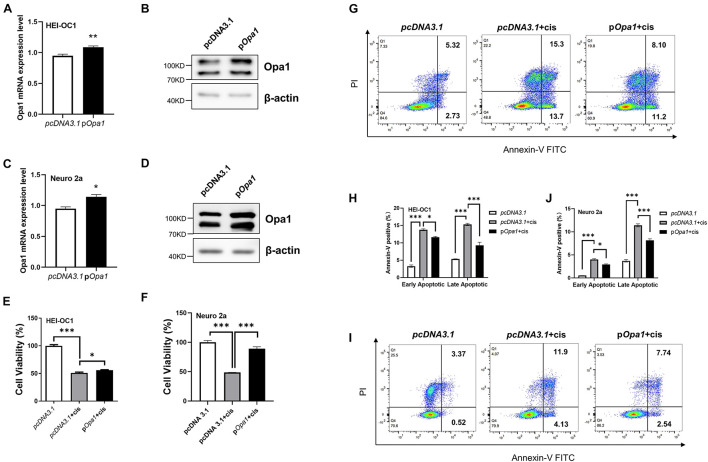
Overexpression of *Opa1* partially restores cisplatin-induced apoptosis in HEI-OC1 and Neuro 2a cells **(A)** Quantification of *Opa1* mRNA expression in HEI-OC1 cells transfected with *Opa1* for 24 h. **p < 0.01 (non-parametric Mann-Whitney *t*-test). **(B)** Representative Western blot images showing the Opa1 protein expression level in HEI-OC1 cells transfected with Opa1 for 48 h. **(C)** Quantification of *Opa1* mRNA expression in Neuro 2a cells transfected with Opa1 for 24 h. *p < 0.05 (non-parametric Mann-Whitney *t*-test). **(D)** Representative Western blot images showing the Opa1 protein expression level in Neuro 2a cells transfected with *Opa1* for 48 h. **(E,F)** CCK-8 was used to assess cell viability in HEI-OC1 **(E)** and Neuro 2a **(F)** cells at 24 h after cisplatin treatment with or without *Opa1* transfection. *p < 0.05, ***p < 0.001 (one-way ANOVA with Dunnett’s multiple comparisons test). **(G)** Representative flow cytometric plots were generated from HEI-OC1 cells stained with Annexin V-FITC/PI to detect apoptosis at 24 h after cisplatin treatment with or without *Opa1* transfection. **(H)** The percentages of early and late apoptotic cells in each group. *p < 0.05, ***p < 0.001 (two-way ANOVA). **(I)** Representative flow cytometric plots were generated from Neuro 2a cells stained with Annexin V-FITC/PI to detect apoptosis at 24 h after cisplatin treatment with or without Opa1 transfection. **(J)** The percentages of early and late apoptotic cells in each group. *p < 0.05, ***p < 0.001 (two-way ANOVA).

To determine if the overexpression of *Opa1* suppresses the apoptosis induced by cisplatin, we first transiently transfected HEI-OC1 and Neuro 2a cells with *Opa1* and examined *Opa1* mRNA and protein expression levels. At 24 h after *Opa1* transfected to HEI-OC1 and Neuro 2a cells, the expression levels of the *Opa1* mRNA (Figures 5A,C) and protein (Figures 5B,D) were significantly elevated. Treatment of both cell lines with 60 μM cisplatin for 24 h caused almost half of the cells to die (Figures 5E,F), but these death rates were diminished significantly in cells transfected with *Opa1* (Figures 5E,F). Furthermore, flow cytometric analysis showed that upregulation of *Opa1* reduced the number of early and late apoptotic cells in HEI-OC1 cells (Figures 5G,H). Opa1 was also found to protect Neuro 2a cells from cisplatin-induced apoptosis (Figures 5I,J).

### Opa1 Protects Against Cisplatin-Induced Hair Cell Death

**FIGURE 6:**
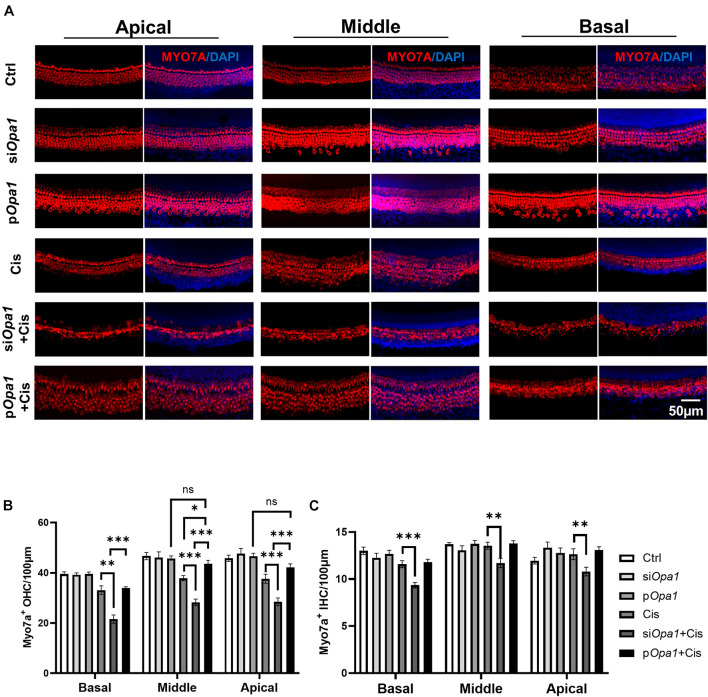
*Opa1* knockdown or overexpression respectively aggravates or partially rescues cisplatin induced hair cell damage in organotypic cochlear cultures. **(A)** Representative confocal images of the apical, middle, and basal regions of organotypic cochlear cultures transfected with si*Opa1* or *Opa1* and treated with 60 μM cisplatin. Hair cells are indicated by MYO7A staining (red) and nuclei are indicated by DAPI staining (blue). **(B,C)** Quantification of IHCs **(B)** and OHCs **(C)** in the apical, middle, and basal turns. *N* = 3–5 per groups. ^∗^*p* < 0.05, ^∗∗^*p* < 0.01, ^∗∗∗^*p* < 0.001 (two-way ANOVA).

Next, we tested the otoprotective potential of Opa1 *in vivo*. Organotypic cochlear cultures were treated with 60 μM cisplatin for 24 h, and hair cell damage was observed in whole-organ inner ear explants. Hair cell morphology and structure were evaluated by immunolabeling of the hair cell marker, myosin 7a. As shown in the Figure 6A, knockdown or overexpressed *Opa1* didn’t affect cochlea morphology. Under cisplatin treatment, the numbers of OHCs were significantly reduced in the apical, middle, and basal turns of the cochlea, but there was no change in the number of IHCs (Figures 6B,C). *Opa1* knockdown followed by cisplatin treatment resulted in more severe cochlear lesions: There were 30% fewer OHCs and 20% fewer IHCs compared to the cisplatin treatment groups (Figures 6B,C). On the contrary, the overexpression of *Opa1* was modestly effective in preventing OHCs death in the middle turn of explants exposed to cisplatin (Figures 6B,C).

## Discussion

Mitochondrial fission and fusion are counterbalancing mechanisms that control the shape, size, and number of organelles. These dynamic processes are also critical in regulating cell death, mitophagy, and organelle distribution (Itoh et al., 2013). In many adherent cell types, such as Hela cells, mitochondria form elongated tubules that continually divide and fuse to form a dynamic interconnecting network (Suen et al., 2008). As shown in Figures 2A,B, we observed elongated and tubular mitochondria in HEI-OC1 cells, then they became highly fragmented upon the loss of Opa1. In Neuro 2a cells, in contrast, fragmented mitochondria were observed by MitoTracker staining even in untreated control cells (data not shown), which is consistent with a previous report that only 30% of mitochondria in this cell line had a length at least twice their width when observed by transmission electron microscopy (Qiao et al., 2017). Because of this, we were unable to observe a change in mitochondrial shape following *Opa1* knockdown in Neuro 2a cells, as assessed by MitoTracker staining. In addition to this difference in mitochondrial shape in the effect of *Opa1* knockdown, HEI-OC1 cells and Neuro 2a cells exhibited differences in the progression of cell death following *Opa1* knockdown: *Opa1* downregulation was associated with significant cell death at 48 h in Neuro 2a cells and at 72 h in HEI-OC1 cells (Figures 3A,B). Moreover, at 24 h after Opa1 depletion, more early apoptotic cells were seen in HEI-OC1 cells, whereas more late apoptotic cells were found in Neuro 2a cells (Figures 3G,H). This difference appeared to reflect the knockdown efficiencies of the two cell lines, indicating that *Opa1* knockdown level is correlated to apoptosis.

Cisplatin is an effective chemotherapeutic agent that is widely used to treat a variety of malignant tumors. In recent years, cisplatin has been reported to affect many different pathways, including cell cycle arrest, apoptosis, proliferation, DNA repair, the TCA cycle, and glycolysis (Pabla and Dong, 2008; Li et al., 2016; Li H. et al., 2018; Gentilin et al., 2019; Liu W. et al., 2019; Zhang et al., 2020; Liu et al., 2021). The anti-tumor effect of cisplatin is mainly due to its ability to interfere with tumor cell proliferation (Wang and Lippard, 2005). Soon after its administration, cisplatin binds to nuclear DNA, where it blocks transcription and induces double-strand breaks leading to cell cycle arrest (Siddik, 2003). As cells from the cochlea are not proliferative, it is thought that mtDNA damage is a more likely cause of cisplatin-induced hearing loss than nuclear DNA damage (Hutchin and Cortopassi, 2000). Hair cells, spiral ganglion neurons, and the stria vascularis have been considered the three major targets of cisplatin ototoxicity (Schacht et al., 2012; He et al., 2019, 2020, 2021; Liu Y. et al., 2019; Zhou et al., 2020; Fu et al., 2021b). Several molecular mechanisms have been proposed as mediators of cisplatin-induced ototoxicity. Cisplatin was reported to stimulate ROS, which in turn triggers inflammatory pathways in the cochlea and promotes apoptotic and necrotic cell death (Rybak et al., 2009; Mukhopadhyay et al., 2012; Qi et al., 2019; Ding et al., 2020; Cheng et al., 2021; Fu et al., 2021a; Lv et al., 2021). As shown in Figure 2 of the present work, Opa1 deletion significantly elevated ROS generation and reduced MMP in both hair cells and neural cells. Cisplatin was reported to decrease *Opa1* mRNA expression in the HK-2 kidney cell line (Choi et al., 2015). We therefore speculated that *Opa1* expression might involve in cisplatin induced hair cell damage. Furthermore, our observation that HEI-OC1 and Neuro2a cells treated with cisplatin exhibited downregulation of *Opa1* transcription and translation level (Figures 4C,D). Meanwhile fragmented mitochondria were observed when HEI-OC1 cells treated with cisplatin (Figure 4E), which may due to the reduction of Opa1. These results indicated Opa1 depletion would exacerbate cisplatin-induced damage.

OPA1 is essential for IMM fusion and plays a prominent role in maintaining the membrane ultrastructure of cristae and the function of mitochondria (Chan, 2020). OPA1 is ubiquitously expressed in all human tissue (Alexander et al., 2000; Delettre et al., 2000). The highest transcript level is found in retina, followed by the brain, testis, heart and skeletal muscle (Alexander et al., 2000).

Though OPA1 is expressed in multiple systems, OPA1 dysfunction diseases are all related to peripheric neuropathy. Furthermore, the rich expression of OPA1 is found in both white and brown adipocytes, and it’s expression elevated during lipid accumulation in adipocytes (Chu et al., 2017). OPA1 has been reported as a dual-specificity A-kinase anchoring protein associated with lipid droplets (Pidoux et al., 2011). At the meantime, many studies reported that Opa1 dysfunction has been involved in muscle mass and cardiac function (Wai et al., 2015; Tezze et al., 2017). Many OPA1 disease mutations have been identified, some of which result in OPA1 haploinsufficiency (Chun and Rizzo, 2017). Mutations in OPA1, particularly truncating mutations, most often manifest as DOA (Cipolat et al., 2004). DOA is characterized by progressive bilateral vision loss, along with hearing loss, myopathy, and peripheral neuropathy (Amati-Bonneau et al., 2008; Hudson et al., 2008). Opa1 dysfunction have been known resulted in RGC neuropathy, increasing the susceptibility of RGCs to apoptosis and vulnerability to oxidative stress (Williams et al., 2010). Given the crucial functions of OPA1, many studies have sought to increase Opa1 levels *in vivo* and *in vitro* as a potential therapeutic approach (Del Dotto et al., 2018). In a transgenic mouse model, a mild increase of the Opa1 isoform 1 protein level was found to protect mice from denervation-induced muscular atrophy, ischemic heart and brain damage, and hepatocellular apoptosis (Varanita et al., 2015). Moreover, *Opa1* overexpression efficiently rescued the phenotypes of two mouse models of defective mitochondrial bioenergetics: Ndufs4^–/–^ and Cox15^sm/sm^ (Civiletto et al., 2015). Overexpression of each of the eight Opa1 isoforms or different constructs encoding isoform 1 of Opa1 in *Opa1*^–/–^ MEFs revealed that any isoform could restore the crista structure, mtDNA abundance, and energetic efficiency independent of the mitochondrial network morphology (Del Dotto et al., 2017). Although it is known that mutation of *OPA1* often leads to hearing loss, it remains unclear how OPA1 is involved in cochlear dysfunction. Our present results indicate that Opa1 plays key roles in maintaining mitochondrial function and preventing apoptosis induced by cisplatin damage. Future work is warranted to assess the potential of OPA1 as a treatment target for cochlear protection and/or repair.

## Conclusion

Our results show that depletion of Opa1 affects the mitochondrial function and cell survival of cochlear cells. We herein report that Opa1 depletion is detrimental to the survival of cisplatin-treated cochlear cells both *in vitro* and *in vivo*, and that *Opa1* overexpression protects cochlear cells against cisplatin-induced ototoxicity in cell lines and in organotypic tissue cultures. Our findings collectively suggest that Opa1 modulates mitochondrial function and is essential for the survival of cisplatin-exposed cochlear cells. In conclusion, our study provides strong cell biological evidence that Opa1 can protect against cisplatin-induced cochlear cell death by enhance mitochondrial function.

## Data Availability Statement

The original contributions presented in the study are included in the article/Supplementary Material, further inquiries can be directed to the corresponding author/s.

## Ethics Statement

The animal study was reviewed and approved by the Ethics Committee of the Shanghai Jiao Tong University School of Medicine.

## Author Contributions

TD designed, supervised the research, analyzed, and interpreted the data. TD and XZ performed the research. TD and ML wrote the manuscript. All authors reviewed the manuscript and discussed the work.

## Conflict of Interest

The authors declare that the research was conducted in the absence of any commercial or financial relationships that could be construed as a potential conflict of interest.

## Publisher’s Note

All claims expressed in this article are solely those of the authors and do not necessarily represent those of their affiliated organizations, or those of the publisher, the editors and the reviewers. Any product that may be evaluated in this article, or claim that may be made by its manufacturer, is not guaranteed or endorsed by the publisher.

## Supplementary Material

Supplementary Figure 1 The uncropped immunoblots for data presented in the Main Figures.
