# Supplementary figures and images for: Opa1 Prevents Apoptosis and Cisplatin-Induced Ototoxicity in Murine Cochleae
Source: Front Cell Dev Biol. 2021 Sep 21;9:744838. doi: 10.3389/fcell.2021.744838 (PMC8490775; doi:10.3389/fcell.2021.744838)

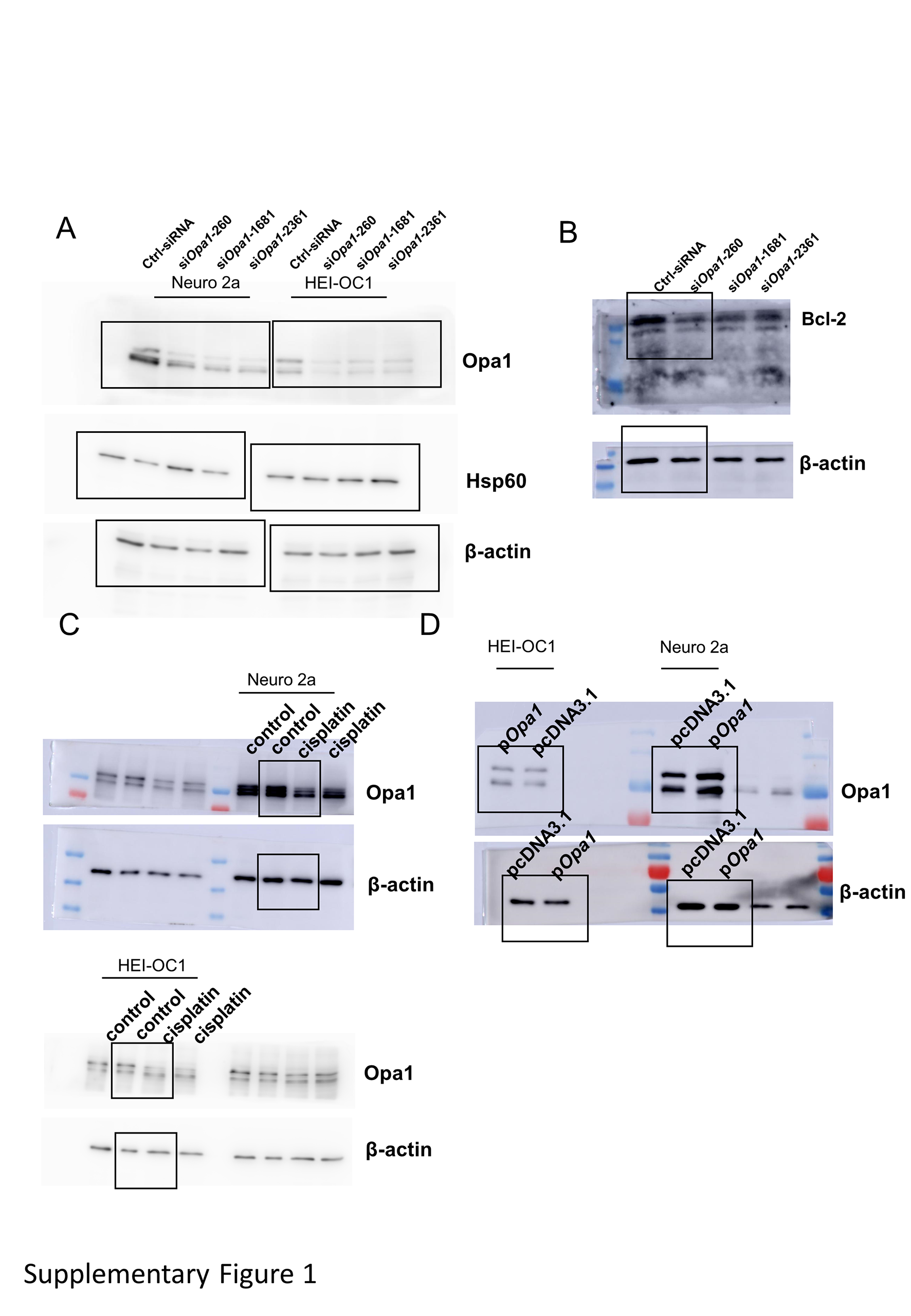

Supplement: Supplementary Figure 1 — The uncropped immunoblots for data presented in the Main Figures. [file Image_1.TIF]
